# Numerical Simulation of Bone Defect Repair Using a Triply Periodic Minimal Surface Scaffold

**DOI:** 10.3390/jfb17050257

**Published:** 2026-05-21

**Authors:** Zhouyang Chen, Haifei Chen, Chuanyong Qu

**Affiliations:** Department of Mechanics, School of Mechanical Engineering, Tianjin University, Tianjin 300350, China; zhouyang_chen@tju.edu.cn (Z.C.); chenhaifei@tju.edu.cn (H.C.)

**Keywords:** triply periodic minimal surface, polylactic acid, numerical simulation, bone tissue engineering

## Abstract

Polylactic acid (PLA) scaffolds with triply periodic minimal surface (TPMS) structures have become ideal scaffolds in the field of bone defect repair due to their good designability, connectivity, biocompatibility, and degradability. However, it is currently difficult to obtain the scaffold degradation rate and osteogenic efficacy from in vivo experiments, making it challenging to provide recommendations for scaffold design. In this study, an algorithm to construct a TPMS scaffold–interfacial layer–tissue three-phase composite model was developed using polylactic acid hydrolysis and bone remodeling as the governing equations to simulate scaffold degradation and tissue osteogenesis behavior under an external mechanical stimulus. This method is based on a numerical calculation framework that can more closely simulate the in vivo environment, characterizing the changes in the overall macroscopic mechanical properties of tissue under the influence of scaffold degradation and tissue osteogenesis. The results confirmed the accelerating effect of mechanical stimulation on scaffold degradation and its promoting effect on new bone formation. Under 10% compressive loading, the Schwarz P representative volume element (RVE) lost 33% of its apparent modulus within initial days, while the lidinoid RVE, despite showing a much higher initial modulus, dropped to only 20% of its initial value over the same period. In addition, the mechanical performance of the fused TPMS RVE was not simply linear, even though the surface equations are combined linearly. These results provide a new method for pre-designing scaffold structures based on numerical simulation results using the finite element simulation.

## 1. Introduction

Trauma, infection, and tumor resection are the primary causes of bone defects [1,2,3]. As a typical biomaterial, bone tissue is most notably characterized by its self-repair capability. However, when the damage exceeds the critical size for bone repair (generally considered to be greater than 2.5 cm in size [4,5], though this may vary depending on individual circumstances and anatomical location), surgical implantation of scaffolds is required for clinical repair [6]. Bone defect repair demands that scaffolds possess excellent biocompatibility to avoid rejection, abundant pores and a large surface area to facilitate cell attachment and blood supply, sufficient mechanical strength to effectively bear loads during the early post-implantation period, and an appropriate degradation rate that matches the rate of new bone ingrowth [7]. The materials currently used for the fabrication of bone defect repair scaffolds can be primarily classified into metals, bioceramics, composites, and polymers. Metallic materials, due to their excellent mechanical properties and bioinertness, were among the earliest materials used for bone defect repair scaffolds [8]. However, the moduli of metallic materials are significantly higher than that of natural bone, which can lead to stress shielding. Bioceramic materials, including hydroxyapatite (HA) and tricalcium phosphate (TCP), have gradually emerged as novel materials for bone repair in recent years owing to their favorable biocompatibility, and showed a promoting effect on bone cell growth in the in vivo experiments of Leukers et al. [9] and the in vitro experiments of White et al. [10]. Nevertheless, the mechanical properties of bioceramic scaffolds are relatively average, making them more suitable for the repair of bone defects in non-load-bearing sites. Composite materials can often combine the advantages of two or more materials to prepare scaffolds with superior performance. Xu et al. [11] fabricated porous iron–hydroxyapatite (Fe–HA) metal matrix composite (MMC) scaffolds using direct ink writing technology, which controlled the degradation rate by controlling the HA content. However, such scaffolds face the challenge of complex preparation processes. Among the various materials for scaffolds mentioned above, PLA scaffolds, which belong to polymers, have been widely applied in the field of bone tissue engineering due to their excellent safety, processability, degradability, and capacity for loading bioactive factors [12,13,14,15,16,17]. In addition to material properties, the structure of a scaffold plays a decisive role in the success of bone regeneration. Studies have shown that key geometric parameters—including porosity, pore size, pore structure, interconnectivity, and surface curvature—critically influence cell adhesion, proliferation, and differentiation, as well as nutrient transport and vascularization [18]. Conventional porous scaffolds with regular geometries (e.g., those fabricated by random porogen methods or with grid or honeycomb structures) have been widely applied due to their simple manufacturing processes and favorable mechanical properties, but often fail to simulate the complex microstructure of natural bone. This limitation hinders their ability to promote effective cell proliferation and bone remodeling. Consequently, the design of bone repair scaffolds has progressively shifted focus toward mathematically defined geometries. Among these, scaffolds based on TPMS are considered particularly promising [19,20,21]. Compared with traditional porous scaffolds, TPMS structures exhibit several near-ideal characteristics for bone tissue engineering: their structure bears a strong resemblance to the microstructure of trabecular bone, a biomimetic feature that facilitates cell recognition, adhesion, and growth; their smooth, continuously curved surfaces simulate the native microenvironment encountered by cells on bone surfaces, guiding migration, alignment, and differentiation, thereby positively influencing the behavior of osteoblasts and vascular endothelial cells; and their fully interconnected pore networks ensure efficient nutrient transport and metabolic waste removal [22,23,24]. More importantly, as TPMS structures are defined by mathematical implicit equations, it is easy to customize scaffolds with specific structures and porosities by adjusting parameters according to the requirements of a patient’s bone defect. Moreover, this approach enables the design of scaffolds with smooth transitions or functionally graded structures, paving the way for the fabrication of personalized implants. However, scaffold degradation and bone remodeling are two interacting and competing processes—their dynamic balance ultimately determines the success of the repair. The scaffold must initially provide sufficient load-bearing ability to support osteogenesis at the early stage, and then degrade at an appropriate rate as tissue regeneration progresses [25,26]. Therefore, a precise understanding of the relationship between scaffold structure, osteogenic ability, and degradation rate is crucial for predicting the outcome of bone defect repair. Early studies on the osteogenesis and degradation behavior of porous scaffolds predominantly utilized animal in vivo experiments and in vitro immersion experiments. The former approach incurred high experimental costs and extended durations [27], encountered ethical constraints, and was prone to variability influenced by differences among subjects. The latter approach faced challenges in simulating in vivo conditions. Consequently, numerical simulation employing the finite element method to examine the evolution of systemic mechanical properties during the coupled process of scaffold degradation and tissue osteogenesis in diverse conditions has emerged as a complementary methodology to experimental methods. Li et al. [28] employed the FEM to investigate the biomechanical properties of Voronoi and TPMS porous scaffolds while also exploring their underlying osteogenic mechanisms through CFD simulations. The results indicated that the superior biomechanical and fluid dynamic characteristics of Voronoi scaffolds are potential reasons for their enhanced biological performance. Pablo et al. [29] developed a model of TPMS scaffolds for repairing large bone defects. This model simulated the bone remodeling process as two concurrent phenomena: cell infiltration governed by Fick’s law and bone formation modulated by local mechanical stimuli. The findings demonstrated that the scaffold effectively supported cell migration into the defect area and promoted tissue formation. Angili et al. [30] performed compression simulations on an RVE of alginate–magnesium oxide composite scaffolds under periodic boundary conditions (PBCs). The results showed that incorporating 20% MgO nanoparticles into pure sodium alginate increased the mechanical strength of the system to 130 MPa. This study aimed to demonstrate the impact of the structure and mechanical properties of different types of TPMS scaffolds on early degradation behavior under external mechanical stimuli and simulate the evolution of scaffold and tissue over time by constructing a three-phase composite material RVE model. The apparent modulus of the RVE and scaffold residual volume at different time points were used to reflect the bone repair and degradation abilities of the scaffold. The coupling mechanism between scaffold degradation and tissue osteogenesis was explored, and the simulation results can be used to guide the design of TPMS scaffolds.

## 2. Materials and Methods

It is important to emphasize that the degradation of a material and the formation of new bone are two dynamically coupled processes that influence each other. On one hand, the degradation of a scaffold leads to changes in its microstructure (porosity, apparent density, etc.) and mechanical properties over time, which directly alter the mechanical environment in the implanted area. On the other hand, according to the principle of functional adaptation in bone remodeling, the intensity and distribution of mechanical stimuli directly regulate the activity of osteoblasts and the deposition of bone tissue. Considering either process in isolation cannot accurately reflect the process of bone regeneration within the scaffold. Therefore, this study required a coupled consideration of bone remodeling and scaffold degradation.

### 2.1. PLA Degradation

When a PLA scaffold is implanted, water molecules in body fluids diffuse into the material and attack the polymer chains, causing chain scission—a process referred to as hydrolysis [31,32]. Hydrolysis reduces the molecular weight of the polymer, and since the mechanical strength of a polymer is directly related to its molecular weight, the degradation kinetics can be described accordingly. Given that the concentration of body fluid remains approximately constant during hydrolysis, the change in molecular weight follows pseudo-first-order kinetics and can be calculated using Equation (1) [32]:

(1) $$\beta (t)=\frac{M_{n}(t)}{M_{n}(0)}=e^{-\lambda_{0}t}$$

where *β*(*t*) is the normalized molecular weight and *M_n_*(0) and *M_n_*(*t*) represent the initial and instantaneous molecular weights, respectively. *λ*_0_ is the degradation constant of the polymer, the value of which depends on its composition. Furthermore, Li et al. [33] observed in their study on the degradation rate of PLGA scaffolds that the application of mechanical stimulation significantly accelerates polymer degradation. To incorporate the effect of mechanical stimulus into the degradation model, Zhurkov et al. [34] proposed a modified degradation rate, and the corresponding normalized molecular weight is expressed as Equation (2):

(2) $$\beta (t)=e^{-\lambda_{0}t}=e^{-\lambda_{\sigma }\frac{A\sigma }{RT}t}$$

where *σ* is the applied external stress, *R* is the universal gas constant, *T* is the temperature in Kelvin, and *A* is the material constant. The relationship between the normalized molecular weight *β*(*t*) and Young’s modulus *E* is given by Equation (3) [31]:

(3) $$E_{S}(t)=\left(E_{S}(0)-E_{T}\right)\frac{e}{e-1}\left(1-e^{-\beta (t)}\right)+E_{T}$$

where *E_S_*(0) is the initial Young’s modulus of the PLA scaffold and *E_T_* is the initial Young’s modulus of the tissue. Although osteogenesis occurs in tissue at subsequent steps, *E_T_* remains constant in the calculation during the update of the scaffold modulus. This is because the effect of osteogenesis on scaffold degradation is already accounted for through the redistribution of the stress field resulting from the increase in tissue modulus.

### 2.2. Bone Remodeling

Bone remodeling is a highly complex physiological process involving cellular activities regulated by various chemical substances. Due to the complexity of the physiological mechanism underlying bone remodeling, which is currently under preliminary exploration, mechanically based bone remodeling models have gained wider application compared to physiological models. The most representative and relatively mature theory is the “mechanostat” proposed by Frost [35]. This theory posits that bone remodeling occurs only when the mechanical stimuli sensed by the bone differ from the steady-state values. The mathematical relationship describing bone remodeling based on this principle can be summarized as Equation (4):

(4) $$\frac{d\rho }{dt}=B\left(S-k\right)$$

where *ρ* represents an indicator for measuring bone mass, which can stand for bone density, porosity, elastic modulus, etc; *B* is the remodeling rate coefficient; *S* represents the external mechanical stimulus; and *k* represents the steady-state reference value. Subsequent studies have revealed that the so-called steady-state reference value is not a precise numerical value, but rather a range, meaning that bone remodeling is triggered only when the external mechanical stimulus significantly deviates from this steady-state reference range. Therefore, Weinas et al. [36] introduced the concept of a “dead zone” and modified Equation (4) into Equation (5):

(5) $$\frac{d\rho }{dt}=B\left[S-\left(1\pm \omega \right)k\right]$$

where *ω* represents the dead-zone range, indicating that no bone remodeling process is triggered when the stimulus value *S* falls within the interval (1 + *ω*)*k*.

The specific mechanical parameter represented by the mechanical stimulus *S* in the numerical governing equations of bone remodeling remains unclear, as the microscale mechanisms by which mechanical factors modify and regulate bone structure and function during the remodeling process are not yet fully understood. Based on the existing literature, three mechanical parameters characterizing bone loading conditions—strain energy density [37,38], equivalent stress [39,40,41], and equivalent strain [42,43]—have been adopted as remodeling stimuli. In this study, equivalent stress was selected as the mechanical parameter to quantify the mechanical stimulus acting on bone, and its governing equation is expressed as follows.

(6) $$\frac{d\rho }{dt}=\left\{\begin{array}{ccc} B\left(\varphi -\left(1+\omega \right)k\right) & if & \varphi >\left(1+\omega \right)k\\ 0 & if & \left(1-\omega \right)k\leq \varphi \leq \left(1+\omega \right)k\\ B\left(\varphi -\left(1-\omega \right)k\right) & if & \varphi <\left(1-\omega \right)k \end{array}\right.$$

The daily stress stimulus *φ* can be expressed as:

(7) $$\varphi ={\left(\sum_{j=1}^{N}n_{j}\sigma^{m}\right)}^{\frac{1}{m}}$$

where *N* is the total number of load cases, *n_j_* is the number of cycles for the *j*-th load case per day, and *m* is the weight factor for the load. The equivalent stress is defined as:

(8) $$\sigma =\sqrt{\frac{1}{2}\left[{\left(\sigma_{1}-\sigma_{2}\right)}^{2}+{\left(\sigma_{2}-\sigma_{3}\right)}^{2}+{\left(\sigma_{3}-\sigma_{1}\right)}^{2}\right]}$$

where *σ*_1_, *σ*_2_, and *σ*_3_ are the principal stress values. The values of the remaining parameters are *B* = 1(g/cm)/(MPa·Time units), *k* = 50 MPa, and *ω* = 0.1 [40]. Additionally, the relationship between the elastic modulus of bone and its apparent density is determined by Equation (9) [44]:

(9) $$E=3790\rho^{3}$$

Therefore, the formula for updating the elastic modulus of tissue during osteogenesis can be expressed as:

(10) $$E_{\left(n\right)}=3790{\left(\sqrt[{3}]{\frac{E_{\left(n-1\right)}}{3790}}+\frac{d\rho }{dt}\right)}^{3}$$

### 2.3. Three-Phase Composite-Material RVE Model

**Figure 1:**
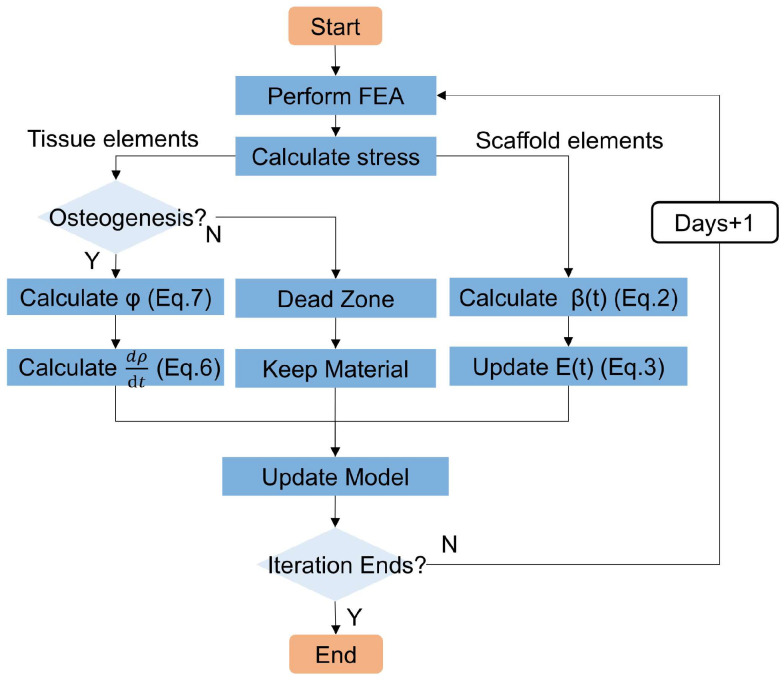
Numerical simulation flowchart.

In this study, an isotropic linear elastic constitutive model was used to describe the damage evolution of the scaffold and the associated changes in tissue material. The detailed numerical simulation procedure is illustrated in Figure 1.

The entire analysis procedure was divided into three steps. In step 1, the displacement load was applied to represent external mechanical stimulation to obtain the stress. Step 2 was subdivided into multiple increments, each increment representing one day, during which the coupled degradation and osteogenesis evolution were simulated. If the equivalent stress of the tissue were within the dead zone, osteogenesis would not be triggered and the material properties would remain unchanged. If the stress exceeded the osteogenesis threshold, the daily stimulation amount and density increment were calculated using Equations (6) and (7). For the scaffold, the normalized relative molecular weight was calculated using Equation (2) and the scaffold modulus calculated using Equation (3). The material properties of the model were updated before the start of the next increment step until the number of cycles reached the set value. In step 3, compression was performed to obtain the σ–ε curve. Mechanical loading was consistently applied throughout degradation simulations. By adjusting the duration of step 2, the mechanical performance of the system after different periods was able to be analyzed.

**Figure 2:**
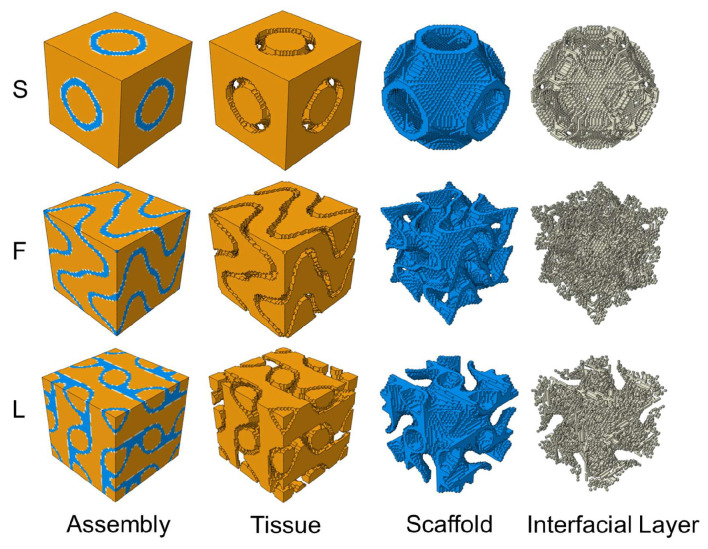
Voxel meshed RVE.

To analyze the osteogenic efficacy and the mechanical properties of the TPMS scaffold, a representative volume element (RVE) was extracted from the periodic structure. This RVE model was composed of a three-phase composite material consisting of the TPMS scaffold, interfacial layer, and tissue, which is generated by Python v3.10, capable of generating user-defined TPMS structures, and allows for adjustments to the scaffold wall thickness and mesh density. Schwarz P is a classic TPMS structure, and Fischer Koch S and lidinoid exhibit structures analogous to trabecular bone. Therefore, these three TPMS types were selected for analysis in this study and are hereafter referred to as S-type, F-type, and L-type scaffolds, respectively. Thickness is uniformly set at 0.3. The implicit functions describing the surfaces of each scaffold are summarized in Table 1, and the porosity of each scaffold was approximately calculated by Equation (11). These three RVE structures are illustrated in Figure 2.

(11) $$1-\frac{V_{Scaffold}}{V_{total}}\times 100\%$$

In order to characterize the volume changes of each part by element number while also balancing computational precision and efficiency accurately, the model was discretized into a cube with a volume of 1 mm^3^ using a hexahedral 8-node reduced integration voxel element with an edge length of 0.02 mm. This discretization resulted in a mesh comprising 50 × 50 × 50 divisions and a total of 125,000 elements. The initial elastic modulus of the PLA scaffold was set to 1350 MPa, the initial elastic modulus of the tissue was defined as 10 MPa and that of the interfacial layer as 50 MPa, and Poisson’s ratios were all set to 0.3 [45]. The remaining parameters are listed in Table 2.

**Figure 3:**
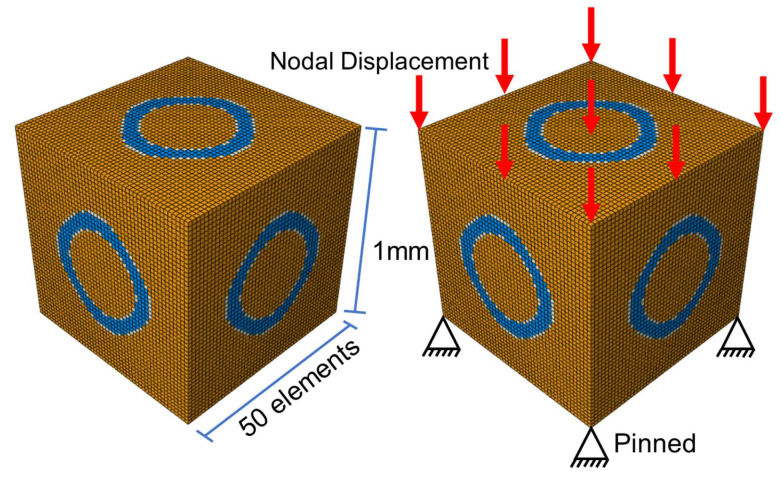
Model size and boundary conditions.

Figure 3 illustrates the finite element model and boundary conditions employed in the degradation simulation. The yellow color represents the tissue, the blue denotes the TPMS scaffold, and the white indicates the interfacial layer between the scaffold and the tissue, which simulates cartilage. In the finite element analysis of TPMS, a 10% compressive strain is commonly used as a typical load for verification and evaluation [46], and thus hinged constraints were applied to all nodes on the bottom surface and a vertical downward displacement load, equivalent to 10%, 15%, and 20% of the RVE edge length, was respectively applied to all nodes on the top surface. The meanings and values of the parameters used in the numerical simulation are listed in Table 3.

## 3. Results

### 3.1. Mechanical Properties of RVE and Scaffold

The RVE model used in this study is a typical composite material. The computation of its apparent modulus was enabled by applying PBCs [49]. As indicated by the mathematical expressions of TPMS structures, any TPMS is centrosymmetric, and thus the RVE mechanical behavior exhibits isotropy, as described by Equation (12).

(12) $$E_{11}=E_{22}=E_{33},\;G_{12}=G_{13}=G_{23}$$

**Figure 4:**
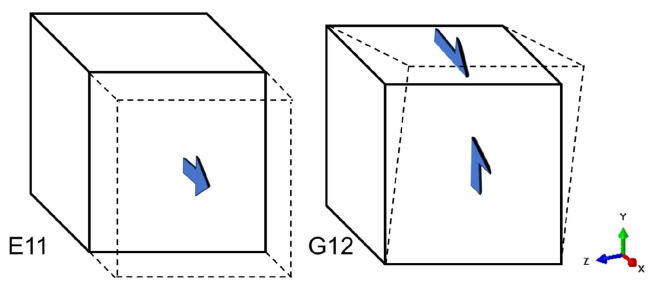
Periodic boundary conditions for calculating apparent modulus.

Appling periodic boundary conditions to the RVE model as illustrated in Figure 4, the apparent modulus *E* and shear modulus *G* can be calculated.

**Figure 5:**
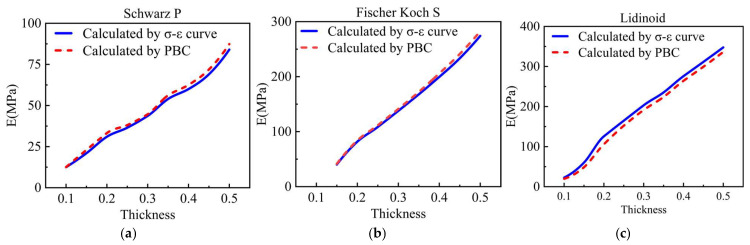
Comparison of the apparent modulus of RVE calculated by applying PBCs and calculated by the stress–strain curve. (**a**) S-type scaffold; (**b**) F-type scaffold; (**c**) L-type scaffold.

As the primary objective of this study was to evaluate the apparent modulus of the RVE after different evolution durations in order to characterize the changes in its macroscopic mechanical properties, and considering the complexity of applying PBCs to the whole model, a simplified approach was adopted in step 3. Specifically, a uniaxial compression simulation was performed on the evolved RVE, and the apparent modulus was calculated from the σ–ε curve. To validate this approach, the results obtained from the σ–ε curve were compared with those computed by applying PBCs (Figure 5). The comparison demonstrated good agreement between the two methods, confirming the validity of this simplified approach.

In the case where the thickness of all the three scaffolds was 0.3, the S-type scaffold exhibited the lowest apparent modulus, approximately 44.68 MPa. In comparison, the F-type scaffold had a higher apparent modulus of approximately 137.78 MPa, while the L-type scaffold had the highest apparent modulus, approximately 191.4 MPa.

**Figure 6:**
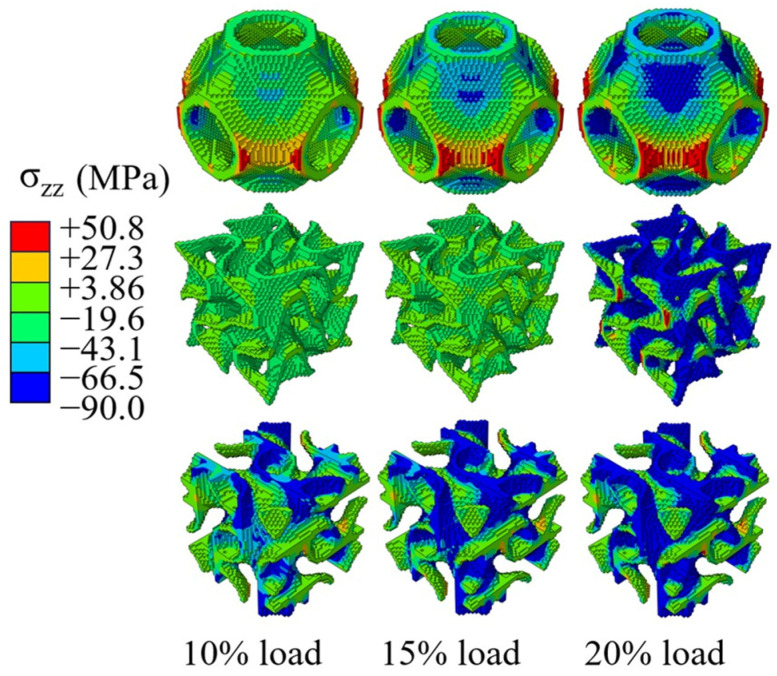
Normal stress distribution in the compression direction under different displacement loading levels.

Figure 6 illustrates the axial normal stress distribution of the three types of scaffold under different displacement loading conditions. In the S type, tensile stress first accumulates at the intersections of the transverse pores. In contrast, the stress distribution in the F type is relatively uniform, while the L type exhibits stress concentration at four centrally symmetric corner points.

### 3.2. Osteogenic and Scaffold Degradation

**Figure 7:**
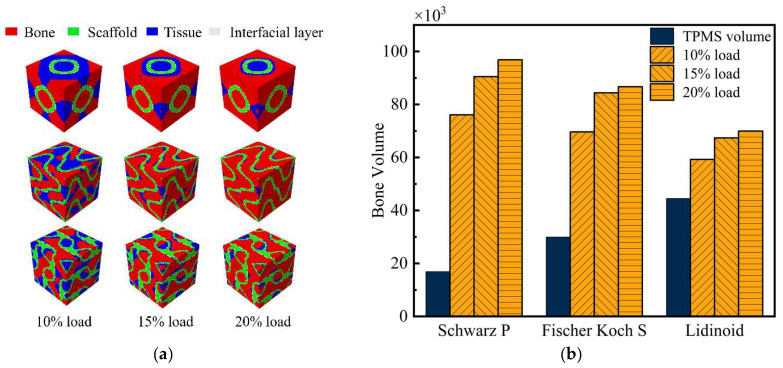
Osteogenic capacity of scaffolds under different loading levels. (**a**) Differences in osteogenic volume distribution of different TPMS scaffolds under three levels of displacement loading; (**b**) differences in osteogenic volume of different TPMS scaffolds under three levels of displacement loading.

The osteogenic capacity of scaffolds under different loading levels is illustrated in Figure 7. It can be observed that an increase in external mechanical stimulation significantly promotes an increase in bone volume. Among the three scaffolds, the S type is the most responsive to external mechanical stimulation, whereas the L type exhibits the least sensitivity to stress stimulation.

**Figure 8:**
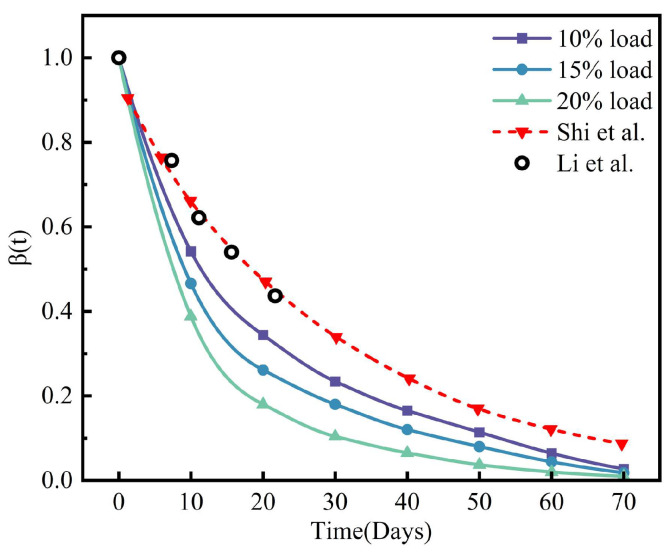
Comparison of the normalized molecular weight between simulation and in vitro degradation experiments without considering osteogenic effects.

To validate the degradation model, this study compared the simulated mean number average molecular weight of the F-type scaffold (without considering osteogenic) with experimental data obtained by Li et al. [47] and the degradation simulation results of a lattice scaffold by Shi et al. [48], as shown in Figure 8.

In the initial stage of degradation, the simulated data aligned well with the results of in vitro experiments, and later the degradation rate of the F-type scaffold exceeded that of the lattice scaffold. In any case, these findings demonstrate the degradation trend exhibited good consistency.

**Figure 9:**
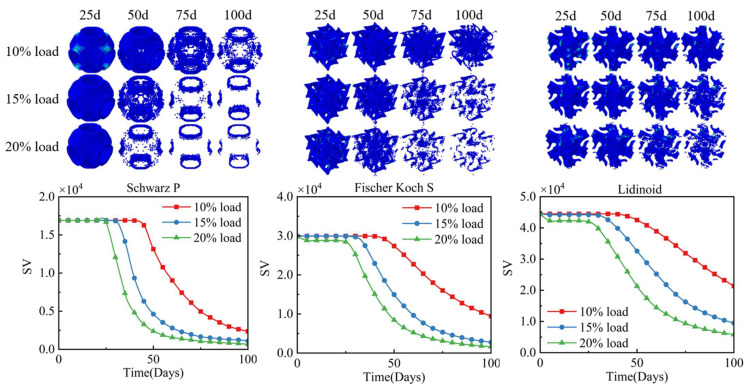
Degradation process of the three types of scaffold under different degrees of load.

**Figure 10:**
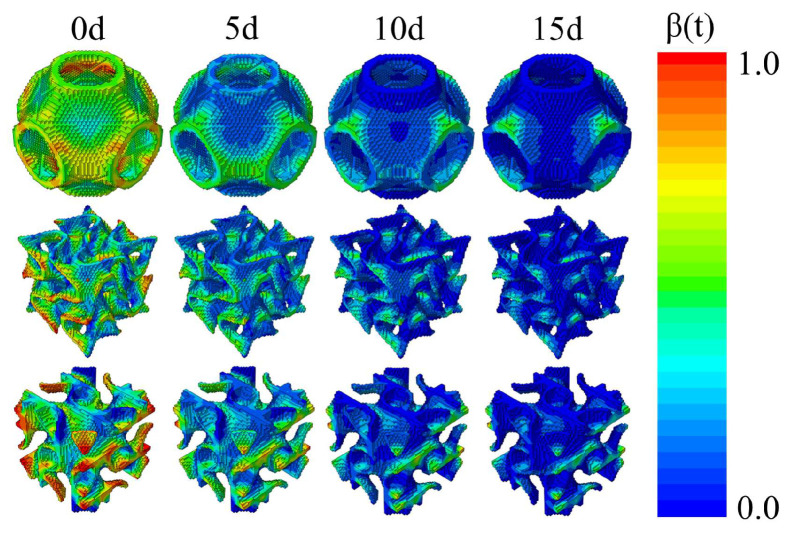
Normalized molecular weight of the scaffolds within the initial 15 days (U3 = −10%).

Comparing the degradation rates of these three scaffolds under different loads, we define the scaffold as fully degraded when its modulus is less than 1% of the initial modulus. Figure 9 and Figure 10 illustrate the residual elements of the TPMS scaffolds, their volume (SV) curves, and normalized relative molecular weight *β*(*t*) of each part at 25 days, 50 days, 75 days, 100 days after implantation under different loads. In the initial stage of degradation, no significant change in scaffold volume was observed, representing the period of damage accumulation. Approximately 25 to 50 days after scaffold implantation, the degradation rate reached its peak, corresponding to the period of polymer chain scission. Under 20% vertical displacement loading, the S-type scaffold had completely degraded in the transverse direction after 25 days, indicating that the scaffold had lost its entire load-bearing capacity at this stage. It was also noted that degradation was slowest at the end regions of the pores, including those aligned with the loading direction. This can be attributed to the elevated stress levels at the junctions of the orthogonal pore networks during the initial degradation phase, which accelerated the onset of degradation in these areas. Subsequently, as osteogenesis progressed, the increase in tissue modulus in the pore end regions induced a stress shielding effect, thereby reducing the mechanical stimulus in these scaffold regions and consequently slowing their degradation.

**Figure 11:**
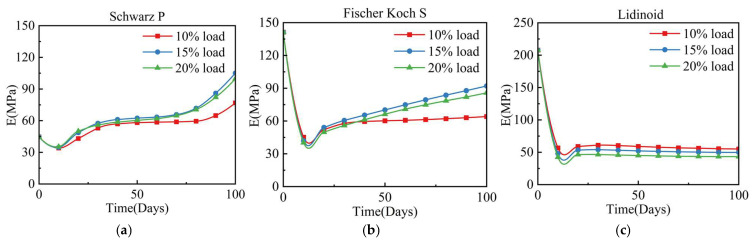
Apparent modulus trajectory under coupled degradation–osteogenesis. (**a**) S-type scaffold; (**b**) F-type scaffold; (**c**) L-type scaffold.

The evolution of the apparent modulus of the RVE over 100 days, as shown in Figure 11, indicates that during the initial 10 days of degradation, the apparent modulus of all three types of RVE decreased. The apparent modulus of the S-type scaffold exhibited a relatively minor decrease, from approximately 45 MPa to 30 MPa. In contrast, RVEs containing the other two TPMS scaffolds underwent a sharp decline, dropping to 20% of their initial apparent modulus within just over 10 days.

### 3.3. Apparent Moduli of Linear Fusion Scaffolds

**Figure 12:**
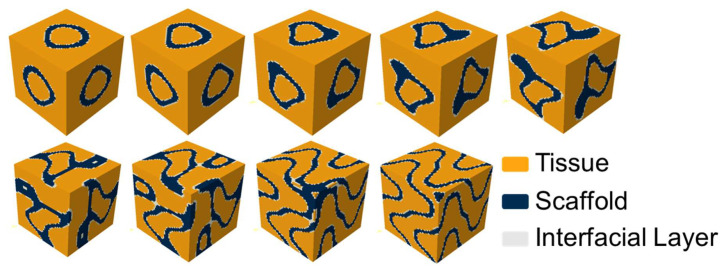
RVEs under different fusion ratios.

The results presented in Section 3.1 indicated that the S-type scaffold demonstrated superior osteogenic efficacy in the later stages, whereas the F-type scaffold and L-type scaffold provided better mechanical support in the early stages. Therefore, this section investigates whether another type of scaffold can be generated through linear fusion of two scaffolds that possesses the combined advantages of both types. This section explores the linear fusion of the S-type scaffold and F-type scaffold. Fusion is achieved through Equation (13):

(13) $$a\left[\cos(x)+\cos(y)+\cos(z)\right]+\;b\left[\cos(2x)\sin(y)\cos(z)+\cos(2y)\sin(z)\cos(x)+\cos(2z)\sin(x)\cos(y)\right]$$

where *a* + *b* = 1 (*a* > 0, *b* > 0). When *a* = 1 or *b* = 1, the structure corresponds to the pure S-type scaffold or pure F-type scaffold, respectively. It can be readily demonstrated from the mathematical expression that regardless of the fusion ratios of the two scaffold types, the periodicity and isotropy of the structure remain unchanged. Figure 12 illustrates the structure of the scaffolds within the RVE for fusion ratios ranging from *a*:*b* = 9:1 to *a*:*b* = 1:9.

**Figure 13:**
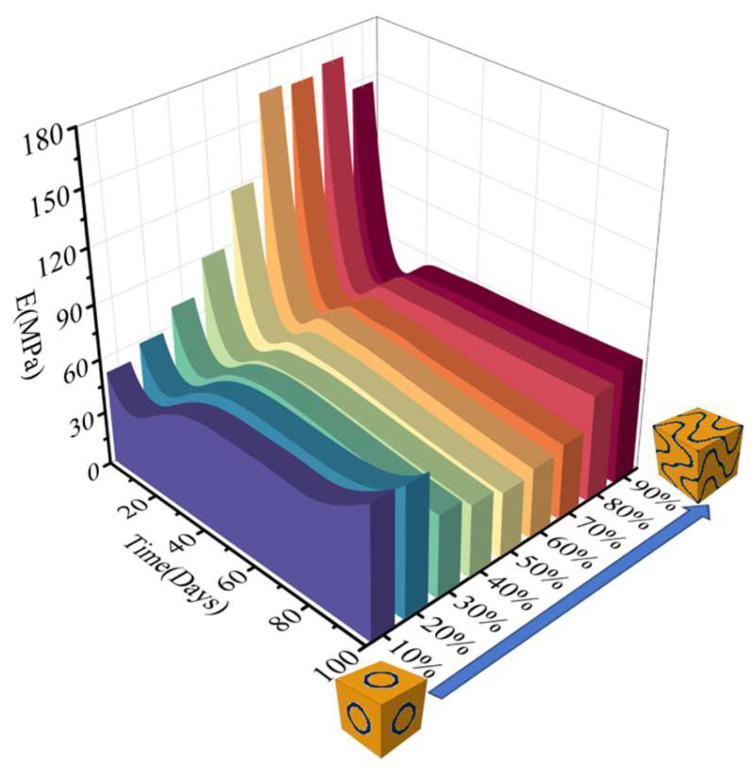
Apparent modulus trajectory of RVE composited by TPMS scaffold in different fusion ratios with coupled degradation–osteogenesis.

Evolution simulations over a period of 100 days were conducted on RVEs with different fusion ratios using 10% compression as the external mechanical stimulus. The changes in apparent modulus over time and the variations in scaffold volume are shown in Figure 13.

In any fusion ratio, the apparent modulus of the RVE always decreases sharply in the initial stage (from day 0), reaching its lowest point around day 20, followed by a slight and brief rebound thereafter, generally exhibiting a V-shaped pattern, with higher values at the beginning and end of the period and a trough in between. When the proportion of a single TPMS structure accounts for less than 80%, the apparent modulus continues to decline after a brief rebound. For an RVE with higher S-type scaffold content, the apparent modulus stabilizes between days 35 and 75 after the short rebound and then increases rapidly. Moreover, the higher the S-type content, the faster the increase. An RVE with higher F-type scaffold content exhibit a similar trend, albeit with a slower rate of increase.

Therefore, regardless of whether the scaffold is S type or F type, as long as the proportion of a single TPMS structure reaches 80% or above, the apparent modulus of the RVE ultimately exhibits a sustained increase after reaching its lowest point. Moreover, the higher the proportion of the single structure, the faster the increase in apparent modulus, with the S-type scaffold demonstrating a more rapid increase in RVE’s apparent modulus compared to the F type. Conversely, when the proportions of the two scaffold types are nearly equal, the apparent modulus paradoxically continues to decline after a brief rebound.

**Figure 14:**
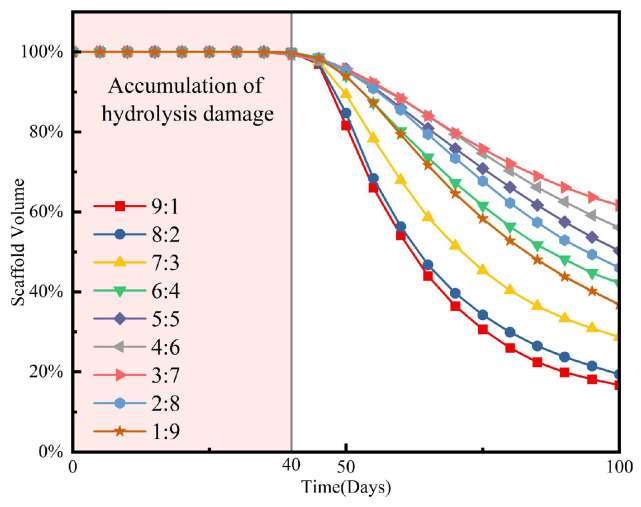
Degradation process of RVE composited by TPMS scaffold in different fusion ratios with coupled degradation–osteogenesis.

A similar phenomenon was observed in the volume change curves shown in Figure 14. Compared to scaffolds with equal fusion ratios, the predominant presence of a single structure accelerated the degradation rate of the scaffold, with an increased proportion of the S-type scaffold leading to a faster degradation rate. Under identical external stimulation, no significant change in scaffold volume occurred during the first 40 days post-implantation, indicating that the onset of degradation is dependent on the magnitude of external stimulation, but independent of the scaffold type.

## 4. Discussion

**Figure 15:**
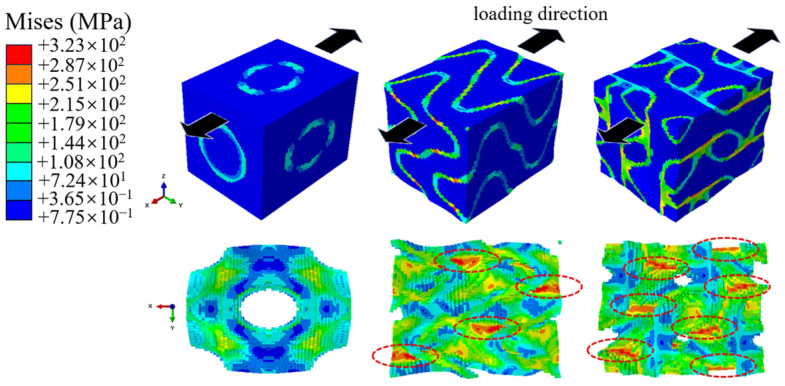
von Mises stress distributions after applying periodic boundary conditions.

In the results presented in Section 3.1, the RVE containing the L-type scaffold exhibited the highest apparent modulus under PBCs. Safa Senaysoy et al. [45] reported that porosity has a significant impact on the apparent modulus of scaffolds, a higher porosity corresponding to a lower apparent modulus. A lower scaffold porosity implies that the scaffold occupies a larger volume in the RVE. Consequently, the proportion of the component with a higher elastic modulus within the system increases, leading to superior overall macroscopic mechanical properties. Among the three types of scaffold, the S type occupied the smallest volume (13.52%), while the L type occupied the largest (35.6%), which is the primary reason for the significant differences in their initial apparent moduli. Furthermore, whether the scaffold structure can form a “force chain” in the loading direction serves as the critical factor in determining whether the apparent modulus can be substantially enhanced. It is well known that in composite materials, optimal load-bearing capacity is achieved when the fibers are aligned with the direction of loading. When a composite material is subjected to load, the load is distributed among the constituent phases according to their respective stiffnesses. If the stiffer phase of the RVE can effectively bear the load, the overall modulus of the system can be significantly enhanced. The distribution of von Mises stress distributions for the RVE in periodic boundary conditions are given in Figure 15. High-stress areas (indicated by red circles) were observed on both the F-type scaffold and L-type scaffold, with the L-type scaffold exhibiting significantly more such areas. This also suggests that in comparison with the S-type scaffold, these two structures are more susceptible to stress concentration.

The apparent moduli of the three RVEs over a 100-day period indicate a decrease in the initial stage after scaffold implantation. This reduction is attributed to the decrease in elastic modulus resulting from scaffold hydrolysis, even though the hydrolytic damage at this stage had not yet led to complete degradation of the scaffold. A similar phenomenon was observed by Yang et al. [50] in their study on the degradation of magnesium alloy scaffolds. In the early stage of osteogenesis, the scaffold modulus decreased with increasing degradation rate. In the later stage, newly formed bone began to reinforce the scaffold structure, eventually offsetting the degradation-induced losses. During the later stage (from 50 to 100 days), the apparent modulus of the RVE containing the S-type scaffold increased more significantly, while the F-type scaffold exhibited a more gradual increase and that of the L-type scaffold tended to stabilize. This is primarily due to the fact that the S type possesses interconnected pores in all three directions, thereby providing favorable conditions for the interconnected growth of bone. In contrast, the structures of the other two scaffolds are more likely to isolate newly formed bone, predisposing the system to non-union of bone and resulting in a relatively lower apparent modulus of the RVE in the later stage (Figure 7). The reason of why F-type scaffold and L-type scaffold exhibit higher apparent modulus and provide stronger load-bearing capacity after implantation is that the scaffolds bear the majority of the load. However, this also results in their rapid degradation in the initial days. At this time, the load-bearing capacity of the scaffold is in a relatively fragile period. Therefore, in the first 3 weeks after implantation, patients should avoid complete weight-bearing or high-intensity activities to prevent collapse of the scaffold. For the F type and L type, rehabilitation plans should be more conservative, such as delaying weight-bearing. For the S type, early functional exercise or gradual weight-bearing rehabilitation strategies can be adopted appropriately; After 40 days, the RVE modulus tends to stabilize or slightly increase, and further increases in exercise intensity can be considered in combination with medical imaging evaluation.

The results in Section 3.3 indicate that the mechanical performance changes of the new scaffold obtained by linearly combining the function of the S type and the F type exhibit significant nonlinearity. When the ratio is close to 1:1, the apparent modulus of the RVE does not show long-term stable growth. Li et al. [51] compared the mechanical properties of Diamond and Schwarz P after fusion. The results showed that the load-bearing performance of the Diamond–Schwarz P model varied nonlinearly under different fusion ratios. In the Schwarz P-dominated model, a 3:7 ratio showed the best load-bearing performance, but the load-bearing performance of the model decreased at ratios of 6:4 and 7:3. This study proposes the concept of “average deflection angle,” which refers to the angle between the direction of each triangular surface of the TPMS model and the direction of the load-bearing force. When the average deflection angle is 90°, the model’s load-bearing performance is optimal. This result is similar to the statement in this study: whether the orientation of structure in scaffold is aligned with the loading direction has a significant impact on the apparent modulus of the RVE. In this study, the new scaffolds obtained by mixing S-type and F-type scaffold at a ratio close to 1:1 may have a smaller average deflection angle, resulting in the decrease in apparent modulus.

This study has several limitations that need to be improved in future work. The first is that assuming degradation occurs under uniform hydrolysis kinetics without considering the local pH changes caused by the accumulation of degradation products and their impact on degradation rate. Secondly, the external stimulus used in bone remodeling algorithms is equivalent stress, and in fact equivalent strain and strain energy density are also considered factors that trigger bone remodeling. Ghassabi et al. [52] conducted similar work using strain energy as an external stimulus. Numerical simulation results showed that the rigid and Schwarz P structures were superior to cubic lattice scaffolds in supporting bone growth and maintaining structural stability. Their study also observed a decrease in the apparent modulus of the RVE within the initial days. Thirdly, this study did not consider the anisotropic adaptation of bone and the effect of fluid shear stress on cell differentiation. Finally, the external mechanical load was simplified as displacement loading in the simulation without considering dynamic changes or multiaxial loading. Despite the above simplifications, the results of this study still capture the core dynamic characteristics of degradation and osteogenesis coupling, qualitatively simulating the trend of RVE apparent modulus changes containing TPMS structures and revealing the feasibility of designing a new type of bone scaffold through linear fusion and predicting its mechanical properties through numerical simulation.

In future research, the introduction of machine learning algorithms will also be of great help in predicting scaffold behavior and bone reconstruction. Hamidreza et al. [53] introduced the guided trajectory distillation machine learning method Osteoflow based on Lyapunov and successfully predicted the repair effect of a mandible after a one-year bone reconstruction cycle. We are confident that machine learning has similar broad application prospects in predicting the relationship between TPMS structural parameters and future mechanical properties. Another important modeling approach is to consider the roles of growth factors, osteoblasts, and osteoclasts in bone remodeling behavior, as proposed by Komarova et al. [54], in order to explore the coupling of external mechanical stimuli and cells at the molecular physicochemical level in this study. Finally, combining professional medical image processing software such as 3D Slicer and Materialise to scan bone-CT slices of patients can more realistically restore bone morphology, thus linking the microlevel research in this article to the macrolevel.

## 5. Conclusions

The numerical framework developed in this study simulates the degradation of PLA scaffolds with a TPMS structure and the evolution of tissue osteogenesis under mechanical stimulation. The competition between hydrolysis-induced stiffness loss of the RVE and stress-driven bone formation determines the long-term mechanical behavior of the scaffold–tissue system and is sensitive to the structure of TPMS scaffolds and external stimuli. The initial load-bearing capacity and long-term recovery ability of the scaffold are related to the type of TPMS: scaffolds with more interconnected networks exhibit a lower initial apparent modulus of RVE, but better recovery in the later stage, while scaffolds capable of forming continuous force chains in the loading direction provide better support in the initial stage, but degrade faster.

The linear fusion of two TPMS scaffolds may exhibit nonlinear mechanical behavior due to negative hybridization effects: the mechanical properties of the RVE containing the hybrid TPMS are weaker than those of the RVE containing either individual TPMS scaffold. Only when the content of a certain TPMS is excessively high can the RVE achieve stable and long-term mechanical recovery.

From the perspective of scaffold design, this work can predict the performance of a scaffold after implantation, including the evolution of structural integrity and load-bearing capacity. It provides a reasonable basis for selecting and designing scaffolds according to specific patient needs, as well as formulating postoperative rehabilitation plans.

## Figures and Tables

**Table 1:** Surface equations of the three types of TPMS scaffold.

TPMS	Equations
S	cos(x)+cos(y)+cos(z)
F	cos(2x)sin(y)cos(z)+cos(2y)sin(z)cos(x)+cos(2z)sin(x)cos(y)
L	$\begin{array}{l} 0.5[\sin(2x)\cos(y)\sin(z)+\sin(2y)\cos(z)\sin(x)+\sin(2z)\cos(x)\sin(y)]-\\ 0.5[\cos(2x)\cos(2y)+\cos(2y)\cos(2z)+\cos(2z)\cos(2x)]+0.15 \end{array}$

**Table 2:** Parameters of the finite element model.

TPMS	Thickness	Element Type	Scaffold Elements	Tissue Elements	Total Elements	Porosity
S	0.3	C3D8R	16,904	103,344	125,000	86.4%
F	0.3	C3D8R	29,944	87,648	125,000	76.04%
L	0.3	C3D8R	44,512	72,344	125,000	64.39%

**Table 3:** Input parameters of simulation.

Parameter	Symbol	Value	Units
Degradation rate constant	*λ*_0_	0.0075 [47]	day^−1^
State change threshold	*β_threshold_*	0.01 [48]	-
Material constant	*A*	22 [47]	J/(mol·K)
Gas constant	*R*	8.314	J/(mol·K)
Temperature	*T*	310	K
Initial modulus of scaffold	*E_S_*	1350	MPa
Initial modulus of tissue	*E_T_*	10	MPa
Poisson’s ratio of scaffold	*ν_S_*	0.3	-
Poisson’s ratio of tissue	*ν_T_*	0.3	-
Number of cycles	*n*	1000	day^−1^
Remodeling threshold	(1 + *ω*)*k*	55	MPa
Remodeling rate coefficient	*B*	1	(g/cm)/(MPa·Time units)

## Data Availability

The raw data supporting the conclusions of this article will be made available by the authors on request.

## Abbreviations

The following abbreviations are used in this manuscript.

PLA	polylactic acid
TPMS	triply periodic minimal surface
RVE	representative volume element
PBCs	periodic boundary conditions
SV	scaffold volume
